# Wind tunnel and numerical data on the ventilation performance of windcatcher with wing wall

**DOI:** 10.1016/j.dib.2016.09.007

**Published:** 2016-09-19

**Authors:** Payam Nejat, John Kaiser Calautit, Muhd Zaimi Abd. Majid, Ben Richard Hughes, Iman Zeynali, Fatemeh Jomehzadeh

**Affiliations:** a Faculty of Civil Engineering, Universiti Teknologi Malaysia, UTM, Skudai, Johor, Malaysia; b Department of Mechanical Engineering, University of Sheffield, Sheffield, UK; c Construction Research Center, Institute of Smart Infrastructure and Innovative Construction, Universiti Teknologi Malaysia, Johor, Malaysia; d Faculty of Engineering, Ferdowsi University of Mashhad, Iran; e Tabadol Dama Gostar Company, Tehran, Iran; f Advanced Building and Environment Research (ABER) Center, Iran

**Keywords:** Windcatcher, Natural ventilation, CFD, Badgir

## Abstract

The data presented in this article were the basis for the study reported in the research articles entitled “Evaluation of a two-sided windcatcher integrated with wing wall (as a new design) and comparison with a conventional windcatcher” (P. Nejat, J.K. Calautit, M.Z.A. Majid, B.R. Hughes, I. Zeynali, F. Jomehzadeh, 2016) [1] which presents the effect of wing wall on the air flow distribution under using the windcatchers as a natural ventilation equipment. Here, we detail the wind tunnel testing and numerical set-up used for obtaining the data on ventilation rates and indoor airflow distribution inside a test room with a two-sided windcatcher and wing wall. Three models were integrated with wing wall angled at 30°, 45° and 60° and another windcatcher was a conventional two-sided device. The computer-aided design (CAD) three-dimensional geometries which were produced using Solid Edge modeler are also included in the data article.

**Specifications Table**

**Table:** 

Subject area	*Environmental Science*
More specific subject area	*Airflow, Computational modelling, Natural ventilation, Wind tunnel*
Type of data	*Tables, Excel files, /graphs, figure, Computer aided design (CAD) geometry*
How data was acquired	*ANSYS and FLUENT 14.5for numerical modelling, Hot-wire anemometer (OMEGA^®^ HHF-SD1) for airflow measurement*
Data format	*Raw, analysed*
Experimental factors	*The wind speed in the 2 m x 1.5 m test section of the wind tunnel was controlled. The three-dimensional windcatcher geometry, domain geometry and boundary conditions were based on actual setup in the wind tunnel test. An atmospheric boundary layer (ABL) flow profile obeying the power law with an exponent of 0.25 was set at inlet. The domain dimensions and position of windcatcher model were specified based on the guidelines of AIJ and COST 732.*
Experimental features	*For the experimental tests, the measurements were carried out in a 1:10 scaled model of a windcatcher with test room. The measurements of supply airflow velocity were performed along points distributed below the windcatcher supply channel. The temperature of the wind tunnel was stabilized before conducting measurements. For the numerical model, Reynolds-averaged Navier-Stokes (RANS) equations were solved using ANSYS and FLUENT14.5. Standard k-e was used as turbulence model.*
Data source location	*Malaysia (experimental data) and UK (numerical data)*
Data accessibility	*Data is with this article*

**Value of the data**

• In order to validate the numerical model, the experimental results of the air flow rate were measured. All of ventilation parameters such as: air flow supply rate, air distribution are compared with the experimental results.
• The data provides a common benchmark which can be used to refine the comparability between the results of other researcher׳s model.
• The presented data could be utilized to assess various turbulence modeling, boundary conditions, mesh types, discretization scheme, steady state and transient simulations.
• The data can be used for CFD user training and contribute to improvement of the precision of modeling of two-sided windcatchers and wing walls.
• The data can be employed to assess various modifications of the two-sided windcatcher with wing wall design.

## 1 Data

**Fig. 1:**
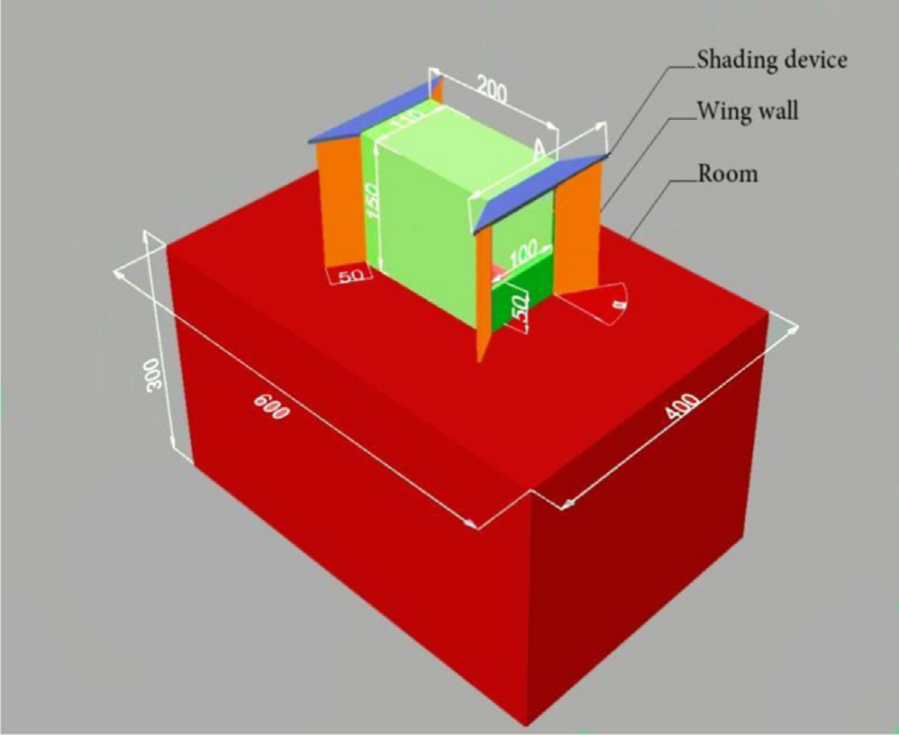
Schematic of windcatcher with anti-short-circuit device (ASCD).

The data presented in this article is based on the experimental results and simulation of the natural ventilation in a two-sided windcatcher device integrated with a wing walls (Fig. 1) which was conducted using wind tunnel testing and numerical modeling [1]. The data used for the investigation of four types of windcatcher configurations: standard two-sided windcatcher (reference model), windcatcher with 30° wing wall, windcatcher with 45° wing wall and windcatcher with 60° wing wall are shared in the article. The CAD files which were created using SolidEdge software are also provided (Supplementary 2.igs files x 4) to save time and effort.

## 2 Design, materials and methods

### 2.1 Experimental design and collection of data

**Fig. 2:**
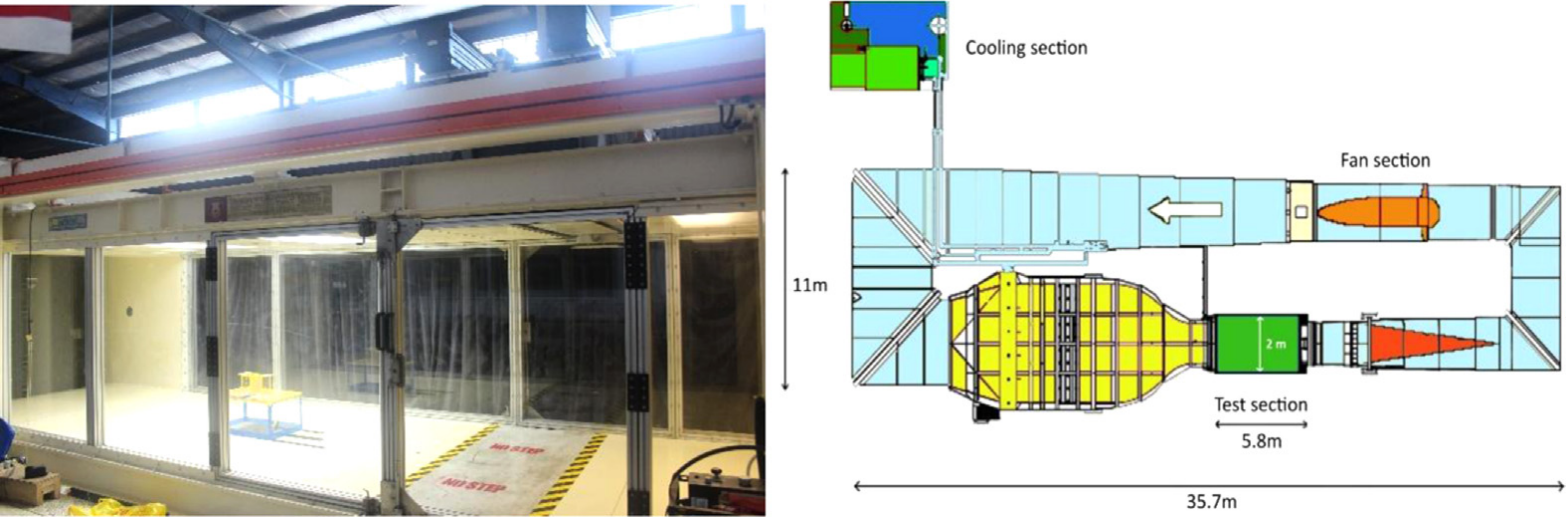
The test section of wind tunnel of University Technology Malaysia and its plan with dimensions.

One of the most equipped and largest wind tunnels in the south East Asia is the Low Speed Wind Tunnel of University Technology Malaysia (UTM-LST) which is also a member of Subsonic Aerodynamics Testing Association. This wind tunnel, which is illustrated in Fig. 2, is a closed-circuit, horizontal return wind tunnel with a rectangular test section of 2 m (W) * 1.5 m (H) * 5.8 m (L) and able to generate a maximum wind speed of 80 m/s in the test section with atmospheric pressure.

**Fig. 3:**
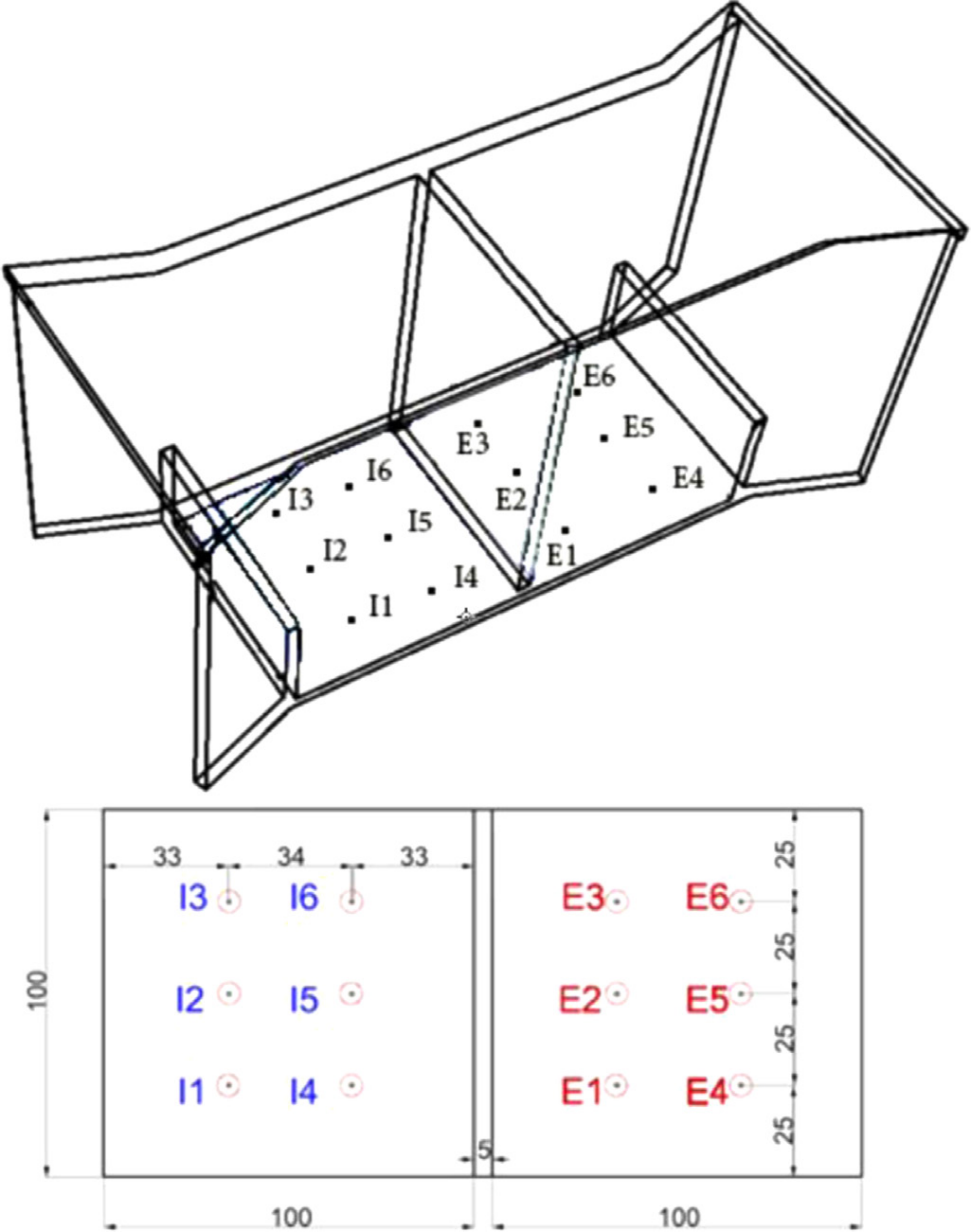
The I and E points in inlet and outlet diffuser of models (all the dimensions are in mm).

In order to evaluate the small scaled experimental model, four different model are produced by 5 mm Plexiglas sheets which are cut by laser with the accuracy of 0.001 mm. Three different angles are suggested four wing walls (30°, 45° and 60°) and a rectangular cuboid with the length of 600 mm and the width of 400 mm is prepaid (see Fig. 1). A 5 mm internal partition is used to separate the inlet and outlet regions. As a base model, the forth model is provided with the windcatcher and without the wing wall. With respect to reference [2], the height of windcatcher was selected 150 mm. In addition, the size of openings and cross-sections of windcatcher were 100 mm by 100 mm [3], [4]. Six different points are chosen to measure the air velocity at the inlet (I_1_ to I_6_) and outlet (E_1_ to E_6_) sections in a symmetric grid parallel and same level to the roof (Fig. 3). The measurement was conducted in Z vertical direction for each point and all data are collected for 1 min.

Air velocity measurement was based on the Constant Temperature Anemometry (CTA) method. The OMEGA^®^ HHF-SD1 is used as an anemometer which can log the data with the resolution of 0.01 m/s and 5% accuracy of reading. The mentioned anemometer could measure the one dimensional flow in two different types: mean value and fluctuating velocity. The dimension of main probe is 1.27 mm length with the diameter of 4 µm.

### 2.2 Numerical design and collection of data

ANSYS14.5 FLUENT CFD tool was used to simulate the streamline of the air flow inside and around the small scaled model. The numerical tool was used to solve the Reynolds averaged Navier–Stokes (RANS) equation which employs Finite Volume Method (FVM) technique. The simulation employed the Semi-Implicit method for pressure-linked equations (SIMPLE) algorithm. Second-order upwind scheme was selected to discretize all the transport equations. Several turbulent model were evaluated and the k-epsilon turbulence model was selected and used for defining the turbulence kinetic energy and dissipation rate within the model. The rationale behind choosing the turbulence model was the findings of previous studies [3], [4], [5], [6], [7], which displayed its capabilities in predicting natural ventilation flows in windcatcher devices. In order to found the optimum mesh size, the selected sizing of the mesh was based on a sensitivity analysis, convergence monitoring and flux balance. The complete meshed model comprised of 8.14 million elements. The profile of the airflow velocity was imposed at the inlet with the streamwise velocity of the approaching flow obeying the power law with an exponent of 0.25 which corresponds to a sub-urban terrain. The simulations were conducted using parallel-processing on a workstation with Intel Xeon 2.1 GHz CPU processor and 16 GB Fully Buffered DDR2. The final results collected based on the maximum iteration without the variation in the fluid dynamic parameters and the numerical calculation were finished when the maximum change in the hydrodynamic parameters are ignorable. The FLUENT post-processing tools is utilized to report the vertices and other plane plotted around the windcatcher and test room.

## Appendix A Supplementary material

**Supplementary 1 Table 1:**Data of the wind tunnel testing of wind catcher with wing wall – This data file presents the measurements of the air velocity in the diffusers of a two-sided windcatcher with no wing wall (reference) and windcatcher with 30°, 45° 60° wing walls.

.

**Supplementary 1 Table 2:**Data for the analysis of ventilation rates based on wind tunnel setup – The data presented in this file are the airflow velocity measurements inside supply and exhaust channels of the windcatcher for different wing wall angles compared against the CFD results.

.

**Supplementary 1 Table 3:**Data for the analysis of supply air velocity – The data presented in this file are the CFD results of the air velocity inside supply diffuser of the windcatcher in different wing wall angles.

.

**Supplementary 1 Table 4:**Data for the analysis of airflow distribution – The data presented in this file are the measurements of the air velocity in the test room (in a horizontal plane) with a windcatcher with different wing wall angles.

.

**Supplementary 1 Table 5:**Data for indoor air quality analysis – The data presented in this file are the measurements of the air velocity in the diffuser of the windcatcher with wing wall compared with windcatcher with no wing wall in different wind speeds, compare the induced air flow rate of windcatcher with wing wall with ASHRAE minimum requirements and finally the air change rate in different wind speeds.

.

**Supplementary 2 (.igs files x 4):**Windcatcher and domain geometry**–** The data files are CAD models of the windcatcher, test room domain and outdoor domain geometry. The models are available in (.IGS) format and can be imported to most CFD tools or platforms such as ANSYS. There are four windcatcher configurations: standard two-sided wind catcher, wind catcher with 30° wing wall, wind catcher with 45° wing wall and wind catcher with 60° wing wall.

.

**Supplementary 2 (.igs files x 4):**Windcatcher and domain geometry**–** The data files are CAD models of the windcatcher, test room domain and outdoor domain geometry. The models are available in (.IGS) format and can be imported to most CFD tools or platforms such as ANSYS. There are four windcatcher configurations: standard two-sided wind catcher, wind catcher with 30° wing wall, wind catcher with 45° wing wall and wind catcher with 60° wing wall.

.

**Supplementary 2 (.igs files x 4):**Windcatcher and domain geometry**–** The data files are CAD models of the windcatcher, test room domain and outdoor domain geometry. The models are available in (.IGS) format and can be imported to most CFD tools or platforms such as ANSYS. There are four windcatcher configurations: standard two-sided wind catcher, wind catcher with 30° wing wall, wind catcher with 45° wing wall and wind catcher with 60° wing wall.

.

**Supplementary 2 (.igs files x 4):**Windcatcher and domain geometry**–** The data files are CAD models of the windcatcher, test room domain and outdoor domain geometry. The models are available in (.IGS) format and can be imported to most CFD tools or platforms such as ANSYS. There are four windcatcher configurations: standard two-sided wind catcher, wind catcher with 30° wing wall, wind catcher with 45° wing wall and wind catcher with 60° wing wall.
